# Supplementary material for: Comprehensive Annotation and Functional Exploration of MicroRNAs in Lettuce
Source: Front Plant Sci. 2021 Dec 24;12:781836. doi: 10.3389/fpls.2021.781836 (PMC8739914; doi:10.3389/fpls.2021.781836)
Supplement: Supplementary file 3 [file Data_Sheet_9.ZIP › GSM2230752.plot/Lsa-miR6118_Lsat_1_v5_gn_2_69520.1_97_TPlot.pdf]

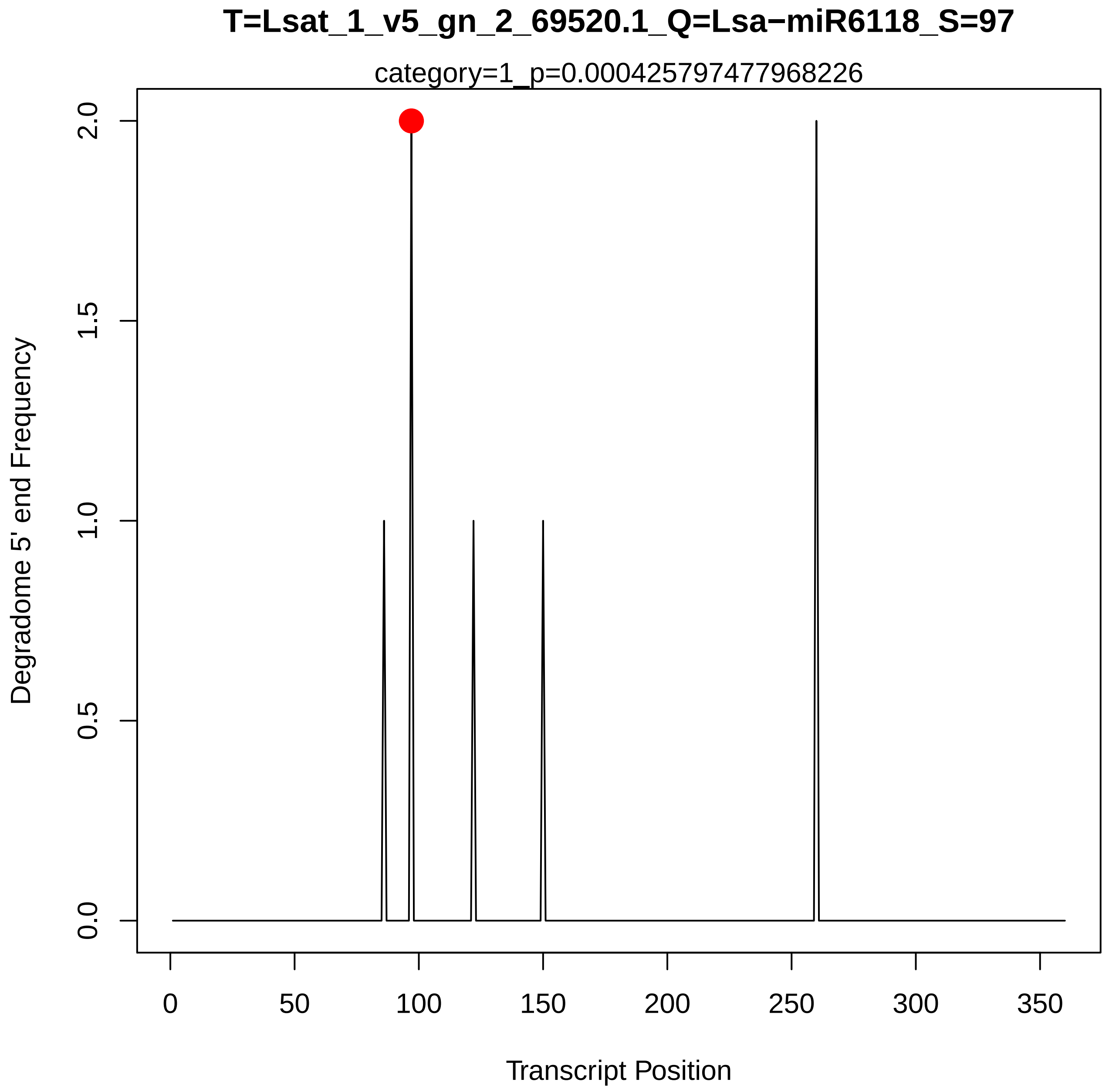
T=Lsat_1_v5_gn_2_69520.1_Q=Lsa−miR6118_S=97
category=1_p=0.000425797477968226
Degradome 5' end Frequency
0.0
0.5
1.0
1.5
2.0
Transcript Position
0
50
100
150
200
250
300
350
